# Cancer and pH Dynamics: Transcriptional Regulation, Proteostasis, and the Need for New Molecular Tools

**DOI:** 10.3390/cancers12102760

**Published:** 2020-09-25

**Authors:** Brandon J. Czowski, Ricardo Romero-Moreno, Keelan J. Trull, Katharine A. White

**Affiliations:** 1 Department of Chemistry and Biochemistry, University of Notre Dame, Notre Dame, IN 46556, USA; bczowski@nd.edu (B.J.C.); rromero2@nd.edu (R.R.-M.); ktrull@nd.edu (K.J.T.); 2 Harper Cancer Research Institute, University of Notre Dame, South Bend, IN 46617, USA

**Keywords:** cancer, pH regulation, transcription, proteostasis, tumorigenesis, biosensors, molecular tools

## Abstract

**Simple Summary:**

As tumors grow, cancer cells must overcome the normal signals designed to keep cell growth in check. Most cancer cells do this by turning off proteins that prevent growth or turning on proteins that stimulate growth through mutation but also through changes in the levels of these proteins inside cells. This review article summarizes recent research that suggests the acidity or basicity (pH) of the environment inside cancer cells may allow cancer cells to specifically stabilize proteins that help them grow and remove proteins that induce cell death. We also discuss new research tools that allow us to measure and manipulate pH in cells to better understand the role pH plays in enhancing cancer growth and progression.

**Abstract:**

An emerging hallmark of cancer cells is dysregulated pH dynamics. Recent work has suggested that dysregulated intracellular pH (pHi) dynamics enable diverse cancer cellular behaviors at the population level, including cell proliferation, cell migration and metastasis, evasion of apoptosis, and metabolic adaptation. However, the molecular mechanisms driving pH-dependent cancer-associated cell behaviors are largely unknown. In this review article, we explore recent literature suggesting pHi dynamics may play a causative role in regulating or reinforcing tumorigenic transcriptional and proteostatic changes at the molecular level, and discuss outcomes on tumorigenesis and tumor heterogeneity. Most of the data we discuss are population-level analyses; lack of single-cell data is driven by a lack of tools to experimentally change pHi with spatiotemporal control. Data is also sparse on how pHi dynamics play out in complex in vivo microenvironments. To address this need, at the end of this review, we cover recent advances for live-cell pHi measurement at single-cell resolution. We also discuss the essential role for tool development in revealing mechanisms by which pHi dynamics drive tumor initiation, progression, and metastasis.

## 1. Introduction

Cancer deaths have not significantly decreased, despite improved therapeutics targeting the genetic basis of cancer [1]. Combating cancer deaths requires addressing tumor heterogeneity [1] and the complex interplay between intracellular and extracellular cues that lead to cancer metastasis [2]. Cancer adaptation and progression is determined in part by genetic diversification and clonal selection under changing tumor properties. To survive, cancer cells must adapt to a dynamic tumor microenvironment [3] including altered metabolism [4], oxygen availability [5], and extracellular matrix composition [6]. Emerging work in the cancer field has refocused efforts on understanding how phenotypic heterogeneity (metabolomic, transcriptomic, and proteomic changes) confer fitness advantages that lead to cancer progression, metastasis, and poor patient prognosis.

**Figure 1:**
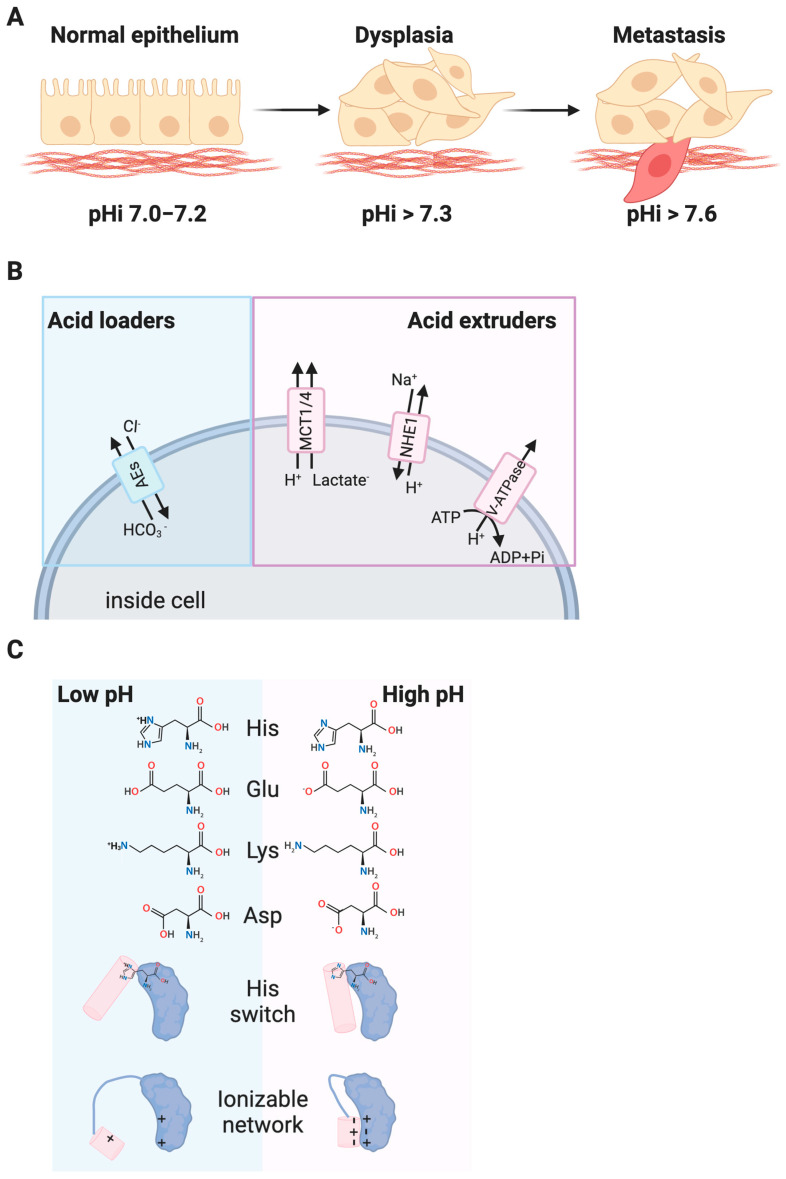
Increased intracellular pH (pHi) in cancer. (**A**) Normal epithelial cells have an intracellular pH (pHi) of 7.0–7.2 while pHi is constitutively increased in dysplastic and metastatic cancer cells. (**B**) Dysregulation of acid loaders (anion exchangers (AE1)) and acid extruders (the sodium proton exchanger (NHE1), monocarboxylate transporters (MCTs), and plasma-membrane resident vacuolar ATPases (V-ATPases) have been linked to the dysregulated pHi in cancer. (**C**) Cellular pH dynamics affect R-group titration of key residues like histidine (His, pKa 6.5) and residues like aspartate (Asp), glutamate (Glu), and lysine (Lys) that can have up- or downshifted pKas depending on protein environment. Changes in protonation of single residues, or networks of ionizable residues can alter protein structure and function.

An emerging hallmark of cancer cells is dysregulated pH dynamics producing an increased intracellular pH (pHi > 7.4) and decreased extracellular pH (pHe < 7.2) relative to normal epithelial cells (pHi ~7.2, pHe ~ 7.4) (Figure 1A). This reversal of the pH gradient is an early event in cellular transformation [7] and can induce dysplasia in the absence of an activated oncogene [8].

Reversal of the pH gradient in cancer is driven by changes in expression and activity of key pH homeostatic regulators (Figure 1B). Acid loaders such as anion exchangers (AEs) [9] lower intracellular pH while acid extruders, such as the sodium proton exchanger NHE1 [10], monocarboxylate transporters (MCTs) [11], and the plasma membrane vacuolar H^+^-ATPase (V-ATPase [12]) raise intracellular pH. While this review will focus on biochemical and cellular effects of changes in intracellular pH, V-ATPase has also been linked to lysosomal dysfunction in cancer [12]. Further elaboration on the role of V-ATPases and organelle-specific pH perturbations can be found in Box 1.

Box 1 **Roles for Vacuolar H^+^-ATPases (V-ATPases) in regulating pH.**

Vacuolar H^+^-ATPases (V-ATPases) represent a family of ATP-dependent proton pumps that contribute to organellar and cytosolic pH. The primary role of V-ATPases is in regulating lysosomal pH, where they establish an acidic luminal pH to enable optimal hydrolase activity. More broadly, V-ATPase involvement in the endolysosomal pathway has clear implications for altering signal transduction and developing chemoresistance in cancer that has previously been reviewed [13]. Recent attention has been brought to increased localization of V-ATPases to the plasma membrane, aiding in maintaining the reversed pH gradient observed with cancer and contributing to cancer cell invasion [14]. This increased localization to the plasma membrane can also drive and reinforce oncogenic Ras signaling [15]. The complex roles of V-ATPases in cancer highlights how dysregulation of both pHi and organellar pH homeostasis may function in concert to enable cancer cell behaviors and disease progression. This work also exemplifies the need for multiplexed tools to image pH in multiple cellular compartments simultaneously.

Recent work has suggested that dysregulated pHi dynamics enable diverse cancer cellular behaviors at the population level including cell proliferation, cell migration and metastasis, evasion of apoptosis, and metabolic adaptation. These pH-associated cancer cell behaviors have been summarized in several recent review articles [16,17,18]. Furthermore, the roles for pHi dynamics in cancer-associated metabolic changes have been extensively covered in recent review article [19,20,21]. However, the molecular mechanisms underlying these pH-dependent cancer-associated cell behaviors are largely unknown. Proteins termed “pH-sensors” have activities, binding affinities, or sub-cellular localization that are regulated by physiological changes in pHi and mediate pH-sensitive cell responses (Figure 1C). Various wild-type pH-sensors have been identified for pH-dependent normal behaviors like directed cell migration (talin [22], cofilin [23]), cell-matrix adhesion (focal adhesion kinase [24]), cell signaling (G-coupled protein receptors [25]), and metabolism (phosphofructokinase [26]). Where they have been molecularly identified, the critical pH sensing residues in these identified pH-sensors are most frequently histidine residues, which can titrate in the physiological pH range. However, the pKas of glutamate (Glu), aspartate (Asp), and lysine (Lys), can be up- [27] or downshifted [28] into the physiological range and networks of ionizable residues can also cooperatively [29,30] titrate to mediate pH-dependent responses within the physiological range. While various pH-sensors responsible for normal pH-sensitive cell behaviors have been identified through careful and extensive biochemical analyses [31], the molecular mechanisms driving pH-sensitive cancer cell behaviors are still largely unknown.

**Figure 2:**
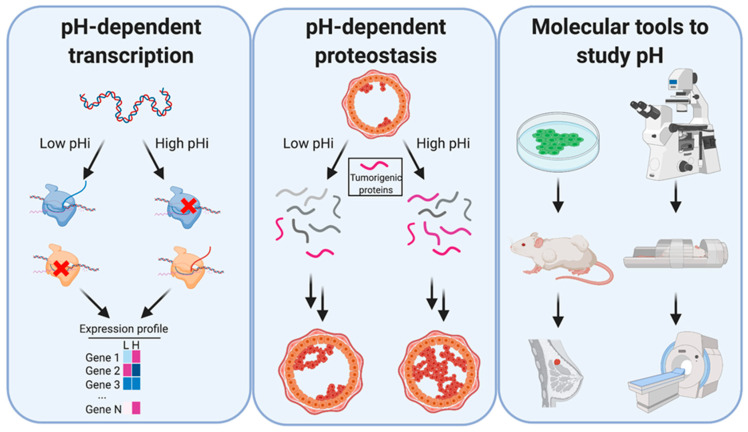
Dysregulated pHi dynamics in cancer can regulate transcription and proteostasis, but better tools are needed to study molecular effects. Left: Altered pH dynamics can affect transcript abundance, where gene 1 transcription is activated by high pHi, gene 2 transcription is activated by low pH, and gene 3 is unaffected. Middle: Altered pH dynamics can also play a role in stabilizing tumorigenic proteins, enabling cancer establishment or progression. Right: Currently, tools exist that allow researchers to study pHi dynamics in living cells by optical microscopy and magnetic resonance imaging (MRI). However, experiments are limited by the constraints of the individual tools, and better tools are required to study the role of pHi dynamics at the cellular, tissue, and organismal level.

In this review article, we will describe and explore recent literature that suggests pHi dynamics may play a causative role in regulating or reinforcing tumorigenic transcriptional and proteostatic changes at the molecular level (Figure 2).

Most of the data discussed herein are population-level analyses; the lack of single-cell data is primarily driven by the lack of tools to experimentally change pHi with spatiotemporal control. Data are also sparse on how pHi dynamics play out in complex in vivo microenvironments. To address this need, at the end of this review, we cover some recent advances for live-cell pHi measurement at single-cell resolution. We also discuss the necessary and essential role that tool development will play in further characterizing the mechanistic role pHi dynamics play in tumor initiation, progression, and metastasis (Figure 2).

## 2. Transcriptional Regulation and pH

**Figure 3:**
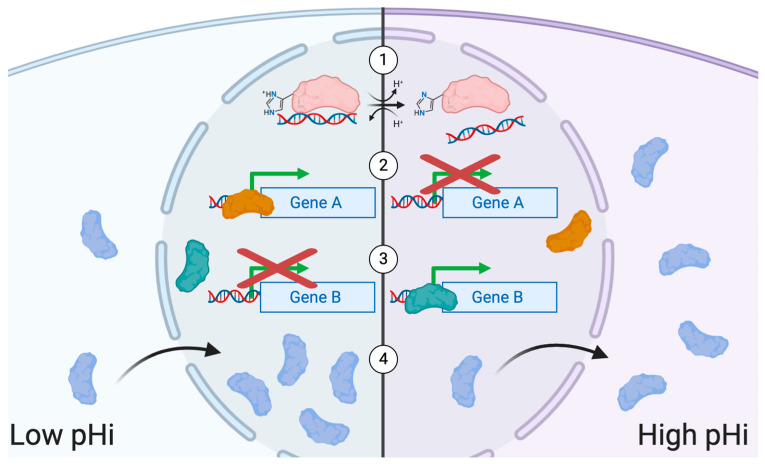
Roles for pHi dynamics in regulating transcription. (**1**) The protonation states of charge-bearing residues can affect the ability of transcription factors to bind DNA under different intracellular pH conditions. (**2**) Transcription factors that actively promote transcription of certain genes at low pHi can lose their ability to promote gene expression at high pHi. (**3**) Conversely, transcription factors that do not promote the transcription of certain genes at low pHi may gain the ability to promote gene expression at high pHi. (**4**) Proteins may exhibit pH-dependent subcellular localization. In the case of transcription factors, moving from the cytoplasm to the nucleus (or vice versa) depending on pHi.

During cancer development, cells undergo significant molecular and phenotypic changes in response to their ever-changing environment. This “cellular plasticity” can be driven by mutations, epigenetic regulation, and transcriptional regulation. Transcriptional regulation is orchestrated by myriad factors including transcription factors (TFs) [32,33], co-regulators of gene expression [34], and transcript lifetime [35,36]. Furthermore, environmental factors such as oxygen availability [37], temperature [38], nutrient availability [39], and pH [40], are emerging drivers of transcriptional changes. Altered transcriptional regulation in cancer can result from dysregulated post-translational modification transcription factors, dysregulated transcription factor or transcriptional regulator binding to DNA, or from mislocalized transcription factors. Importantly, there are demonstrated roles for pHi dynamics in altering each of these transcriptional regulation pathways (Figure 3). In this section of the review, we will address the role of pH in driving changes in gene expression and ultimately conferring fitness advantages to cancer cells.

Notably, it has been shown that pHe [41,42] and pHi [43,44] changes can drive alterations in the gene expression profiles of cancer cells. For example, as tumors grow, oxygen becomes scarce and lactate dehydrogenase A (LDHA) is upregulated as cancer cells shift to aerobic glycolysis generating lactate as a by-product [45]. This increase in lactate production acidifies the cellular environment and has been shown to modulate cancer cell migration and invasion [46] as well as upregulating production of interleukin-8 (IL-8) and vascular endothelial growth factor (VEGF) [45], two well-characterized pro-angiogenic factors [47,48]. Similarly, rapid alkalinization of the cytosol is observed during cancer development [10] from significant increases in activity of the sodium proton exchanger NHE1 [49,50]. High activity of NHE1 is associated with upregulation of the matrix metalloproteinase (MMP)14 [43,44] which plays an important role in cancer cell invasion [51]. Furthermore, treatment with cariporide, a specific NHE1 inhibitor [52], was shown to decrease expression of MMP14 leading to decreased invasion of cancer cells [43,44]. These results suggest that pHi and pHe dynamics function in concert to regulate cancer cell behaviors; however, more research is needed to reveal specific causative roles of each.

Dynamic pH can also directly regulate transcription factors to drive phenotypic plasticity in cancer. For example, acidic culture conditions can induce nuclear localization of the Sterol Regulatory Element-binding protein 2 (SREB2), driving the transcription of twelve pH-responsive genes including acetyl-CoA synthetase 2 (ACSS2), 3-hydroxy-3-methylglutaryl-CoA synthase 1(HMGCS1), farnesyl diphosphate farnesyltransferase (FDFT1), and low density lipoprotein receptor (LDLR) [53] that contribute to invasion [54] (ACSS2), proliferation [55] (FDFT1), increased growth rates [56] (HMGCS1), and advanced tumor grades [57] (LDLR). Further, all nine cancers analyzed by Kondo et al. displayed lower overall survival in patients with high expression of the twelve pH-responsive genes, reinforcing the role that pH may play in cancer establishment and progression through metabolic alterations in response to environment [53]. This study exemplifies how the acidic microenvironment can induce expression of genes advantageous to cancer cells by driving transcriptional changes independent of genomic variation.

In addition to extracellular pH, pHi has been shown to control the subcellular localization of the transcriptional effector, Smad5 [58]. Fang and colleagues showed that alkalinized pHi induced cytoplasmic Smad5 accumulation and accelerated glycolytic flux whereas acidic pHi induced nuclear localization and expression of Smad5 target genes [58]. Additionally, it was also shown that Smad5 plays a crucial role in maintaining the cellular bioenergetic homeostasis by regulating hexokinase 1 (HK1) [58], a rate-limiting enzyme in glycolysis [4]. The subcellular distribution of HK1 has also been shown to relocate from outer mitochondrial membrane (OMM) to the cytosol in acidic conditions and return to the OMM under basic pH in a glioma cell line [59]. These results open the possibility of exploring pHi to address the energetic vulnerabilities of cancer cells by controlling the behavior of these and other metabolic regulators [60].

It has been shown that some somatic mutations may confer a pH-sensitivity to the mutated protein [61]. Importantly, certain amino acid substitutions are significantly overrepresented in cancer [62] and it has been proposed that the cancer mutational landscape is in part shaped by the fitness advantage provided by the pH-sensitive behavior acquired by these somatic mutations [61]. A notable example is the tumor suppressor p53, a protein mutated in roughly half of all cancers [63]. A point mutation in p53 (R273H) can confer pH-dependent function where at high pHi, p53-R273H will show decreased DNA binding and decreased transcriptional activity [64]. This results in a faulty programmed cell death response that can be reverted by decreasing the pHi of cancer cells to reestablish apoptotic responses [64]. Interestingly, Arg273 is the most commonly mutated amino acid in p53 and is crucial for DNA binding. This suggests that this gained pH-sensitive behavior in p53 could be acting as a selective pressure in cancer cells.

Additionally, future work investigating point mutations in other transcription factors may reveal more examples of adaptive gain in pH-sensitive DNA binding. One such possibility is point mutations in the transcription factor FOXP2, that have been shown to play a role in stabilization of DNA binding, where loss of Arg leads to disease [42]. Interestingly, Arg > Isoleucine and His > Tyrosine mutations are also prevalent in the zinc finger domain of several TF in three different types of cancer [65]. These studies highlight how point mutations could be conferring a pH-sensitive behavior to cancer cells. It is possible that altering the amino acids responsible for stabilizing DNA binding will be the subject of future studies utilizing pHi as a therapeutic opportunity to combat cells with dysregulated gene expression profiles.

Taken together, these examples demonstrate that disrupted pH dynamics can drive transcription factor localization or activity and lead to alterations in transcript abundance. Moreover, these data suggest environmental cues such as pH could be key drivers of the heterogeneous acquisition of adaptive fitness advantages in cancer cells. Recent work by Persi et al. has determined that pH-dependent vulnerabilities exist in cancer cell metabolism [60], suggesting a potential therapeutic window of synthetic lethality by inhibiting ion exchangers. This strengthens the evidence that lowering pHi in cancer cells may be therapeutically beneficial, provided we can find a biomarker of increased pHi in tumors.

## 3. Heterogeneity and pHi

As cancer progresses, heterogeneous subpopulations of cancer cells arise with different phenotypic [66] and molecular signatures [67]. Transcriptional heterogeneity in tumors can arise as a response to environmental conditions including pH [68]. Importantly, transcriptional heterogeneity can confer an array of fitness advantages to individual cells that contribute to a higher likelihood of cancer cell proliferation, survival, metastasis, or therapy resistance [64,69].

While hypoxia [70] and metabolic changes [71] have been described as factors that influence transcriptional regulation, little is known about how pH contributes to tumor heterogeneity or whether pHi is a sufficient regulator of gene expression in cancer cells. Furthermore, pH-sensitive cancer-associated phenotypes have often been attributed solely to the effects of pHe [72,73], despite pHi dysregulation being a key driver of altered pHe [74]. For example, transcriptional changes inside cells have been attributed to acidification of pHe [68], but that acidification was experimentally driven by inducing intracellular hypoxia, which also changes pHi [75]. These data suggest a link between extracellular and intracellular pH environments and a role for pHi as a driver of microenvironment remodeling and tumor phenotypic heterogeneity.

In addition to pHi potentially driving microenvironment heterogeneity, recent work shows that pHi may itself be heterogeneous. For example, when cells were selected at distinct pHi levels, their resulting daughter cells had heterogeneous pHi [76]. This suggests not only that pHi distribution may be a stochastic contributor to tumor heterogeneity, but that pHi may be a biomarker for more complex phenotypic heterogeneity markers like stemness, metabolic adaptation, or mesenchymal phenotype [76]. Therefore, it is imperative to study how pHi contributes to fitness advantages in subpopulations within a tumor and to develop therapies that target these cells with pH-dependent vulnerabilities.

## 4. Relationship between Transcript and Protein Abundance

Following the central dogma of biology, the amount of mRNA transcripts should be directly correlated to the amount of protein it encodes. However, studies of ovarian [77], colorectal [78], and prostate cancer [79] show that mRNA transcript levels are poor indicators of protein abundance. This establishes the importance of proteomic analysis in addition to genetic and transcriptomic analyses when diagnosing patients. For example, discordance between mRNA transcript and protein abundance was identified for the tumor suppressor p53 in both breast and colon cancer, where comparable transcript levels produced varying amounts of protein [80]. Similarly, a study showed the oncogenic transcription factor forkhead box protein M1 (FOXM1) is stabilized by overexpression of a deubiquitinating enzyme (USP21); this prevents ubiquitin-mediated degradation and induces expression of proliferative genes [81]. These data support the hypothesis that dysregulation of proteostasis could confer a fitness advantage independently of genomic or transcriptomic changes. Previous efforts have shown that combining transcriptomic data with proteomic analysis allows for better grouping of disease signatures [79], demonstrating that understanding post-translational regulation of protein abundance is crucial for disease diagnosis and treatment.

## 5. Tumorigenesis, Proteostasis, and pHi

**Figure 4:**
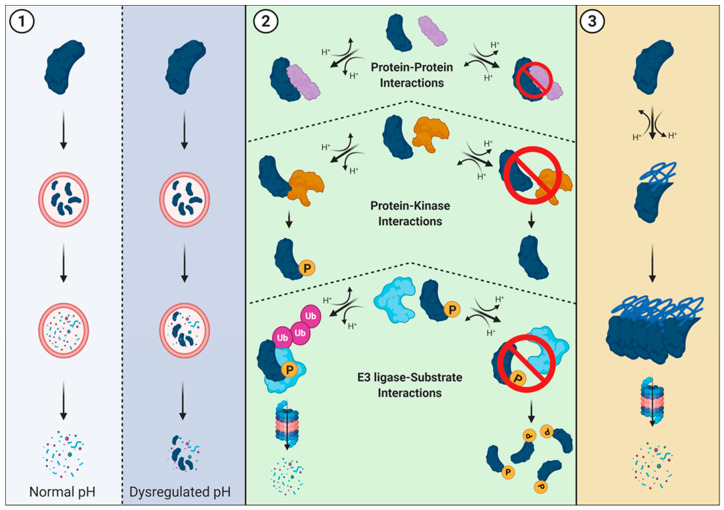
Roles for pH dynamics in regulating proteostasis. (**1**) Lysosomal-mediated degradation. Lysosomes have an acidic pH (~4.5–5.0) that allows for optimal protease activity and protein degradation. However, dysregulation of pH within the cytosol (and subsequent lysosomal pH changes) can result in failed or partial degradation. (**2**) Overview of protein-protein interactions required for proteasome-mediated degradation. If pH-sensitive residues play critical roles in protein interaction interfaces, dysregulated pH may prevent (or enhance) an interaction from occurring. When the pH-dependent interaction occurs between a kinase and substrate, dysregulated pH may alter phosphorylation, and potential downstream signaling for protein-kinase interactions. Similarly, when the pH-dependent interaction occurs between an E3 ubiquitin ligase and its substrate, dysregulated pH may alter ubiquitination and protein degradation. (**3**) Aggregate clearance is the process of removing proteins that have aggregated. Since pH dynamics can also alter the three-dimensional structure of proteins and cause complete misfolding or localized disorder, pH-dependent aggregation is another potential way pH regulates proteostasis.

Dysregulation of the synthesis-degradation axis alters protein abundance and can confer fitness advantages to cells (For review: [82,83]). Fitness advantages in cancer can be conferred by the stabilization of oncogenes and destabilization of tumor suppressors leading to tumorigenic cell behaviors. Altered proteostasis in cancer can result from lysosomal dysfunction, alterations in the ubiquitin-proteosome system, or the clearance of aggregated or misfolded proteins following stress response. There are roles for pHi dynamics in altering each of these proteostatic pathways (Figure 4).

Many pHi-dependent tumorigenic behaviors, such as proliferation, epithelial-to-mesenchymal transition (EMT), and metabolic reprogramming have been shown to be driven by proteostatic changes. For example, stabilization of the oncogene Myoferlin [84] enables tumor growth and angiogenesis through VEGF-secretion in pancreatic cancer [85], promotes migration and EMT through epidermal growth factor receptor (EGFR) recycling in breast cancer [86] and supports oxidative phosphorylation by retaining mitochondrial integrity in colon cancer [87]. Additionally, the transcriptional regulator bromodomain-containing protein 4 (BRD4) had an increased abundance in colon cancer that promoted growth and invasion independently of its transcriptional targets, Myc and B-cell lymphoma 2 (BCL2), by stabilizing acetylated Snail and promoting proliferation through a post-translational regulatory mechanism [88]. Finally, cells cultured in acidic environments showed increased transcript levels and protein abundance of autophagy regulators, providing a mechanism to reduce dependence on environmental nutrient availability while providing the biomass required for growth and proliferation. Collectively, these examples demonstrate how dysregulation of protein abundance at the post-translational level promotes a variety of cancer fitness advantages.

## 6. Proteasome-Mediated Degradation and pHi

Proteasome-mediated degradation is regulated by phosphorylation then ubiquitination, where ubiquitin targets proteins for degradation. Highlighting the importance of degradation motif recognition, a recent analysis of new and known degradation motifs determined that nearly 10% of driver mutations in cancer occur within degrons of substrates or substrate recognition interfaces of E3 ligases [89]. Thus, a population of driver mutations may be contributing to tumorigenesis by dysregulating protein abundance.

The role of pHi dynamics in protein stability has been exemplified in a recent study showing β-catenin functioning as a pH-sensor with decreased stability at increased pHi [90]. A histidine in the destruction motif is responsible for this pH-dependent stability, as the protonation state of that histidine determines if β-catenin is recognized and ubiquitinated by the E3 ubiquitin ligase, β-transducin repeat containing protein (β-TRCP). At increased pHi, decreased cytoplasmic β-catenin stability and abundance may potentially drive the loss of cell-cell junctions in EMT and initiate the metastatic cascade. An emerging concept in the field is that protonation events can be considered post-translational modifications [31]. The pH-dependent stability of β-catenin is just one example of how a titratable residue can mediate global changes in protein function and lifetime. Importantly, this result also suggests that other β-TRCP targets with a conserved histidine in the destruction motif could be sensitive to dynamic pHi, but this has not yet been assessed. More broadly, pH-sensitive binding of either wild-type or mutant E3 ligases and their wild-type or mutant substrates is an unexplored avenue for understanding the role of pHi dynamics in dysregulation of protein abundance.

## 7. Roles for Proteostasis in Tumorigenesis

Overabundance of proteins like Myc and Cyclin E are associated with cancer cell proliferation and cell cycle progression, respectively. Additionally, Ras and Raf are members of the mitogen-activated protein kinase (MAPK) pathway and constitutive activation results in uncontrolled growth signaling in cancer. However, the abundance of these proteins has never been studied in the context of pHi. We next discuss each protein individually, highlighting key experiments that suggest pH-dependent protein stability may enable their tumorigenic function.

Myc is a cancer-associated transcription factor that is targeted for proteasome-mediated degradation by the E3 ligase, Fbxw7. This E3 ligase requires three arginine residues (Arg465, Arg479, and Arg505) for substrate recognition and binding [91], with 29% of Fbxw7 cancer-associated mutations occurring at Arg465 [92]. One of the most frequent Fbxw7 mutations (R465H) replaces a non-titratable Arg with a titratable His residue [93]. This suggests the hypothesis that the Myc-Fbxw7 interaction requires a positively charged residue (arginine) and may become pH-sensitive when that residue is mutated to a titratable histidine. This could produce pH-sensitive degradation of Myc, with Myc being stabilized specifically at the increased pHi of cancer where His465 of Fbxw7 is more likely to be deprotonated. Importantly, the potential impact of charge-changing mutations on protein activity highlights the need to understand the role of these mutations in the context of pHi.

Cyclin E is a kinase that progresses cells through the G1-S transition into DNA synthesis, where dysregulated abundance may result in genomic instability. Previous reports showed constitutive activation of Ras or Raf increased cyclin E abundance, suggesting MAPK activation is sufficient to prevent ubiquitination of cyclin E [94]. The authors argued that Ras activity functions as a rheostat to modulate cyclin E stability by interfering with Fbw7-mediated degradation. It was later shown that B-Raf directly associates with and increases the activity of NHE1, leading to pHi alkanization [95]. Taken together, these results suggest Ras/Raf activation leads to an increased pHi that could modulate the protein-protein interactions required for targeted degradation of cyclin E.

Framing the results from previous studies in the context of pH dynamics reveals a potential role for pH in proteostasis-mediated tumorigenesis. For example, a mutation in either an oncogene (Myc) or tumor suppressor (Fbxw7) may invoke pH-sensitivity that was previously overlooked in protein abundance dynamics. Alternatively, activation of NHE1 and resulting intracellular alkalinization may increase kinase activity to regulate protein degradation pathways and reinforce tumorigenesis. Degradation-associated signaling cues and kinase activation may also be mediated through pHi changes, as we propose for Cyclin E, where activation of NHE1 and resulting intracellular alkalinization increases kinase activity to dysregulate protein degradation pathways and reinforce tumorigenesis. However, it is still unclear how specific molecular events and microenvironment cues may drive dysregulation of proteostasis. While studies involving β-catenin have highlighted pHi as a regulator of protein degradation, more work needs to be done to validate the other examples—Myc and Cyclin E—proposed here.

## 8. Tool Development and New Horizons

Correlations can be made between pHi and transcriptional and proteostatic changes, but we lack studies that conclusively show whether pHi is a common driver of these changes that lead to the acquisition of cancer fitness advantages. Importantly, initiating these mechanistic studies requires improved tools to accurately measure and specifically manipulate pHi for transcriptomic or proteomic analysis. In this section of the review, we will discuss the tools and techniques used to measure and manipulate pHi in current studies, as well as the improvements that need to be made to current methodologies to reveal the complex role of pHi dynamics on transcriptional and proteomic changes in cancer.

## 9. Tools to Measure pHi

The ability to accurately measure pH inside living cells is critical to understanding pHi dynamics during cellular processes and the effect they have on cellular behaviors. Doing so requires tools that can measure absolute pH values with high spatial and temporal resolution. Ideally, these tools can be used to measure live, single-cell pHi with minimal damage to the cell. Please refer to Table 1 for a summary of the features of the tools described below.

Early methods of pHi measurement include the use of patch clamp techniques to analyze pHi [96]. While these methods were initially groundbreaking in enabling quantitative pHi measurement, they are technically laborious, non-physiological, and detrimental to the cell. Thus, there was a clear need for techniques that enabled the measurement of pHi with high spatiotemporal resolution while being compatible with live cell measurements at a single-cell and population level.

The advent of molecular tools to measure pH in living cells helped fill this gap. These tools are capable of measuring pH with high spatiotemporal resolution, decreased technical complexity, and increased reproducibility. There are several groups of molecular tools that have been developed for the measurement of pHi including fluorescent dyes [109], ionic liquids [100], and fluorescent proteins [110]. Using these tools, pHi can be measured at the single-cell level as well as across the entire population. However, one caveat of using these tools is the need for standardization in each experiment. Molecular tools are more sensitive to variations in their environment and therefore must be standardized in each new system or experiment with solutions of known pH, which is not necessary when using direct patch clamp measurements.

Fluorescent pH-sensitive dyes such as the green fluorescent BCECF (2’,7’-Bis-(2-Carboxyethyl)-5-(and-6)-Carboxyfluorescein, Acetoxymethyl Ester) [97], and the red fluorescent SNARF (Seminaphtharhodafluor) [98] have been widely used to accurately measure pHi in cultured cells. These dyes enable quantitative pHi measurement and can be targeted to specific subcellular locations, such as the lysosomes [111], or mitochondria [112]. However, both BCECF and SNARF produce mild cytotoxic effects and photobleach quickly making long-term imaging of pHi during various cell behaviors difficult [109].

Work with other synthetic reporters has recently made improvements such that they are more compatible with long time-scale in vivo work. For example, indole heptamethine cyanine dyes represent another class of pH-sensitive dyes that are useful for tumor and tissue measurements due to their near infrared fluorescence properties; however, they suffer from poor stability and dim fluorescence [99]. Ionic liquids have also recently been developed as pH-sensitive tools compatible with live cell and in vivo imaging [100]. The work by Gao and colleagues demonstrated that ionic liquids can be used to accurately measure pHi decreases caused by drug-induced acidification and hypoxia [100]. The ionic liquids produced little cytotoxic effect over six hours, making them compatible with cell-based experiments. These tools do suffer from relatively low quantum yields and brightness limiting tissue penetration and thus compatibility with in vivo experiments [100]. Furthermore, these tools are non-linear for reporting pHi above 7.4–7.5, making them less applicable for studying intracellular alkanization events [100]. Development of a suite of ionic liquids with shifted pKas and improved brightness may result in them becoming a less toxic alternative to fluorescent dyes that are also compatible with in vivo studies.

Developing fluorescent, protein-based tools to measure pHi is transformative because it allows for stable expression of a sensor that can be easily targeted to subcellular compartments. Most green fluorescent proteins (GFPs) are natively sensitive to pH with pKas around 6.0 [113]. The fluorescent protein pHluorin is a ratiometric GFP variant with mutations that upshift the pKa to enable pHi-measurement in the physiological range [101]. Subsequent improvements produced pHluorin2, a brighter ratiometric variant [104], and super ecliptic pHluorin, a significantly brighter intensiometric variant [101]. Furthermore, pHluorin and its derivatives can be easily targeted and mutated to accurately measure pH in various subcellular compartments. Targeted versions of pHluorin have been used to measure pH in the lysosomes (pH 4.5–6.5) [114], Golgi network (pH 6.0–6.7) [115], endoplasmic reticulum (pH 7.2–7.5) [116], and mitochondria (pH 6.1–8.5) [117]. Such tools, including the litmus-body, a fusion between pHluorin and a nanobody, allow for pH measurement at various targeted cellular locations, expanding the sensing capabilities of pHluorin [118]. Major caveats associated with ratiometric pHluorin include its dim intensity and required blue wavelength stimulation, which has low penetration in live tissue.

To combat some of the issues with pHluorin, red-shifted fluorescent proteins (RFP) that function as pH biosensors have been recently developed, including pHred [105], pHuji [107], and more recently mCherry variants [108,119]. The mCherry EA mutant is a bright ratiometric pH sensor that has been used in combination with pHluorin to measure pH simultaneously in both the cytosol and mitochondria [108]. While red-shifted fluorescence does improve tissue and tumor imaging compatibility, RFP-based pHi sensors suffer from low brightness and significant aggregation, which make quantification more challenging than their GFP-based counterparts. Thus, there is still a need to develop better tools for measuring intracellular pH that are non-toxic, bright, compatible with deep tissue imaging, and easily quantifiable with high spatial and temporal resolution.

## 10. Tools to Manipulate pHi

The ability to manipulate pHi allows us to determine the role pH plays as a potential initiator or driver of tumorigenic cell behaviors. To reiterate, the ideal pHi manipulation tool must have high spatial and temporal resolution, minimal off-target or cell-toxic effects, should be capable of cue-dependent responses and be reversible.

Some of the more recently developed tools focus on various sources of cue-dependent pH manipulations. NMR instruments have been shown to be capable of inducing pH changes in solution [120]. Some examples include small molecule photoacids, which release a proton upon photo-uncaging with UV light [121]. While these tools are promising candidates for pHi manipulation, they have unfortunately not yet been used in live cell experiments and more work will be needed to determine their usefulness as tools in this context.

Current techniques for manipulating pHi often involve disrupting native cellular pH homeostasis mechanisms, such as knock-down or inhibition of specific proton transporters. However, these methods suffer from widespread off-target effects and poor spatial and temporal resolution. Temporal resolution could be improved with transient single-cell knockdown and both spatial and temporal resolution could be improved with photo-uncaging of inhibitors or pH alkanization agents.

More promising spatiotemporal pHi manipulation tool development can be seen in optogenetic tools. While light-activated proton pumps, such as archaerhodopsin, have mainly been used to silence neuronal firing, they have recently been used to alter pHi in cells to monitor gap junction connectivity [122]. However, these tools require near-continuous photoactivation to maintain pH changes, which interferes with the ability to perform experiments on longer timescales. Another issue with using unidirectional proton pump tools is that they also change membrane potential, which has been shown to affect cell behaviors [123]. Next-generation optogenetic tools that require less intense red-shifted light with tunable off-rate kinetics would be transformative for expanding the pHi-manipulation toolbox.

Current tools to measure (dyes, fluorescent proteins) and manipulate (gene knock down, inhibitors, optogenetics) pHi have been useful in establishing our understanding of how pHi functions within the realm of cancer development and progression. However, there is a need for improved tools capable of measuring pHi inside of tissue and animals accurately and with low toxicity. Such tools will allow the study of pHi dynamics of cancer cells within the native tumor microenvironment with minimal perturbation. New tools for pHi manipulation with decreased cytotoxic effects, specific pHi manipulation, and precise control will improve studies to investigate pHi as a driving force in cancer.

## 11. Conclusions

It is clear that phenotypic and genotypic heterogeneity function in concert with microenvironment pressures to create a perfect storm of cancer survival, metastasis, resistance, and evolution. Key to improving patient outcomes is a better understanding of molecular mechanisms driving these cancer hallmarks. Cancer genomics is advancing at an incredible rate, with innovations in single-cell DNA sequencing [124], four-dimensional (4D) genome mapping [125], and ribosome profiling [126]. Genetic sequencing has been revolutionary in the classification and origins of cancer, and organoid drug screening is a potentially transformative approach for screening therapeutics prior to administering to a living patient [127]. However, with the exception of a few superstars such as imatinib for chronic myelogenous leukemia (CML) [128], targeted molecular therapeutics have not been able to consistently overcome issues of tumor heterogeneity and clonal adaptation and selection [129].

The work summarized in this review suggest the tantalizing possibility that tumor pHi may be a biomarker of more complex and difficult to measure markers of tumor heterogeneity such as cancer metabolism, cancer stem cells, or epithelial vs. mesenchymal phenotypes. The work described above suggests that the cell biological and biochemical effects of increased pHi in cancer correlate with and perhaps reinforce transcriptional and proteostatic changes associated with cancer. However, several recent papers that note off-target effects of key pHi manipulating drugs [130,131] have begun to call into question the previous causative role of cytosolic pH in driving tumorigenic phenotypes at the population level. Indeed, the work in this field so far has been crippled by the lack of tools to spatiotemporally manipulate and measure pHi across multiple cancer model systems (cell lines, organoids, and in vivo models) to determine the roles of pHi dynamics across biological scales. If we are to be successful as a field in establishing causative links between pHi dynamics, single-cell behaviors, and tumor behaviors, we need to reinvest and refocus on developing versatile and specific tools to measure and manipulate pHi in real time and that are adaptable to single-cell and in vivo imaging.

As this review shows, our current understanding of pH-sensitive proteins and cell behaviors has been built from decades of slow and careful one-by-one studies. With better tools, we can validate the literature results as well as access previously intractable experiments that explore the role of pHi in regulating global transcriptomic and proteostatic changes. This will allow us to identify pH-sensitive nodes in these cancer-associated pathways that might be therapeutically targetable for limiting tumor progression. Furthermore, quantitative pHi measurements across cancer model systems could alone revolutionize the way clinicians think about targeting pHi-dependent cancer fitness advantages.

## Figures and Tables

**Table 1:** Comparison of Tools for Measuring Intracellular pH.

Tool	In Vivo Compatibility	Cytotoxicity	Long-Term Measurements	Spatial Resolution	Brightness	Quantitative	Requires Standardization
Patch Clamp [96]	Incompatible	high	minutes	Single Cell	NA	Yes	No
BCECF [97]	cell-based	mild	minutes-hours	Subcellular	mid	Yes	Yes
SNARF [98]	cell-based	mild	minutes-hours	Subcellular	mid	Yes	Yes
Indole Heptamethine Cyanine Dyes [99]	cell-based	mild	minutes	Subcellular	low	Yes	Yes
Ionic Liquids [100]	cell-based	low	hours	Subcellular	low	Yes	Yes
pHluorin [101]	cell-based, some tissue	low	hours	Subcellular, targetable	low	Yes	Yes
SuperEclipticpHluorin [101,102]	cell-based, some tissue	low	hours	Subcellular, targetable	high	No	Yes
pHluorin-mCherry [103]	cell-based, some tissue	low	hours	Subcellular, targetable	high	Yes	Yes
pHluorin 2 [104]	cell-based, some tissue	low	hours	Subcellular, targetable	mid	Yes	Yes
pHred [105,106]	cell-based, deeper tissue	low-mild	hours	Subcellular, targetable	low	Yes	Yes
pHuji [107]	cell-based, deeper tissue	low-mild	hours	Subcellular, targetable	low	No	Yes
mCherry EA-mutant [108]	cell-based, deeper tissue	low-mild	hours	Subcellular, targetable	low	Yes	Yes
